# Cochlear immunology and its therapeutic revolution

**DOI:** 10.3389/fimmu.2025.1666224

**Published:** 2025-11-11

**Authors:** Ting Liu, Ruoyun He, Ye Tian, Weibang Chen, Xiaoyu Liu, Lei Zhu, Fengxiang Cao, Zizhong Yu, Xueming Cui, Peng Wang, Yingqi Liu, ZiYing Zheng, Wenxiong Gao, Junmin Huang, Hongying Luo, Lu Chen, Ziqian Bi, Junhao Song, Tianyang Wang, Chia Xin Liang, Junfeng Hao, Han Wang, Chunjie Tian

**Affiliations:** 1 Department of Otorhinolaryngology, Affiliated Hospital of Guangdong Medical University, Zhanjiang, Guangdong, China; 2 Department of Nephrology, National Clinical Key Specialty Construction Program (2023), Institute of Nephrology, Guangdong Provincial Key Laboratory of Autophagy and Major Chronic Non-communicable Diseases, Key Laboratory of Prevention and Management of Chronic Kidney Disease of Zhanjiang City, Affiliated Hospital of Guangdong Medical University, Zhanjiang, China; 3 Faculty of Information Technology, Beijing University of Technology, Beijing, China; 4 Department of Computer Science, China Agricultural University, Beijing, China; 5 School of Advanced Technology, Xi’an Jiaotong-Liverpool University, Suzhou, Jiangsu, China; 6 JTB Technology Corp., Tainan, Taiwan; 7 Department of Family Medicine, Shengjing Hospital of China Medical University, Shenyang, Liaoning, China

**Keywords:** cochlear immunology, blood-labyrinth barrier, sensorineural hearing loss, autoimmune inner ear disease, noise-induced hearing loss, immunomodulation, immunotherapy

## Abstract

Over the past five years, cochlear immunology has experienced a paradigm shift, challenging the long-held perception of the inner ear as an “immune-privileged” site. Our review consolidates recent advancements that elucidate the cochlea’s intricate local immune system, comprising resident macrophages, Tlymphocytes, and dendritic cells, in conjunction with the regulatory blood-labyrinth barrier. We investigate how immune dysregulation contributes to various auditory disorders, including autoimmune inner ear disease, inflammatory responses to cochlear implantation, noise-induced hearing loss, and age-related hearing loss. The review critically assesses therapeutic strategies, encompassing both traditional immunosuppressants and innovative immunomodulatory approaches, as well as interventions targeting fundamental aging pathways. Significant research gaps are highlighted, including the need for reliable biomarkers, a deeper understanding of immune cell heterogeneity, and the development of enhanced drug delivery systems. These advancements present promising opportunities for the development of targeted treatments for immune-mediated hearing loss, with the potential to revolutionize the clinical management of these conditions.

## 1 Introduction

The cochlea, a marvel of biological engineering, serves as the peripheral organ of hearing, responsible for converting sound waves into electrical signals sent to the brain for interpretation (1). The inner ear, including the cochlea, has long been considered an “immune-privileged” site, an assumption primarily based on the blood-labyrinth barrier (BLB), a tightly regulated physiological seal, and the relatively low concentrations of immunoglobulins present in its fluid (2). This perceived isolation suggests that the cochlea remains unaffected by peripheral immune activity.

Recent advances over the past decade, especially during the last five years (2020–2025), have drastically transformed our understanding of cochlear immunology. The cochlea is now understood to harbor a complex and active local immune system rather than being an immunologically inert bystander (2). Research has revealed resident populations of immune cells and demonstrated that the cochlea, particularly its mesenchymal areas, such as the lateral wall, can be a significant site of inflammation and immune activity. This shifting perspective highlights the importance of local immune regulation in maintaining cochlear health, as well as its role in various pathogenic conditions. Understanding the cochlea as an organ with “actively regulated” immunity opens fresh directions for understanding disease pathogenesis, from seeing it as just “privileged”. This complex view implies that therapeutic approaches should aim not only on stopping immune cell entrance but also on changing the local immune responses already present in the cochlea.

Hearing loss is a common worldwide health concern, affecting a significant portion of the population. Current estimates suggest that approximately 20% of individuals worldwide experience some degree of hearing loss, with 5% suffering disabling hearing loss. Most of these cases are categorized as sensorineural hearing loss (SNHL), a condition resulting from damage to, or loss of, the neurosensory structures within the inner ear, including the hair cells and auditory neurons.

The growing discipline of cochlear immunology is of great clinical relevance, as immune dysregulation is increasingly linked to the pathogenesis of several types of SNHL. Among these are disorders including Age-Related Hearing Loss (ARHL), Noise-Induced Hearing Loss (NIHL), Autoimmune Inner Ear Disease (AIED), and inflammatory responses triggered by cochlear implantation (CI) (3). Most importantly, immune-mediated SNHL is among the few types of hearing loss for which suitable and timely therapeutic intervention could cause reversal. This potential emphasizes the necessity to unravel cochlear immunology and design targeted therapies that can modulate these mechanisms to either preserve or restore auditory function.

This review aims to synthesize and evaluate the key research findings published over the past five years that have advanced our understanding of cochlear immunology. Importantly, we focus on clarifying the cellular and molecular components of the cochlear immune system, investigating how immune dysregulation contributes to common cochlear pathologies, and outlining the advancements made in developing and improving therapeutic approaches. Aimed at reducing immune-mediated damage and so promoting auditory health, these techniques range from conventional immunosuppressive treatments to new immunomodulatory approaches and regenerative therapies.

## 2 The cochlear immune system: a contemporary perspective

The long-held understanding of the cochlea as an immune-privileged site, isolated from systemic immune responses, has been significantly altered. Recent studies have highlighted the existence of a dynamic local immune system, comprising structural barriers and resident immune cells, that actively maintains homeostasis and responds to damage or infection.

### 2.1 The Blood-Labyrinth Barrier: dynamic gatekeeper and immune modulator

The BLB functions as a critical physiological interface, isolating the delicate inner ear environment from the systemic circulation. Primarily found in the spiral ligament and the capillaries of the stria vascularis, the BLB is composed of specialized endothelial cells interconnected by tight junctions, pericytes, and a continuous basement membrane (3). Its fundamental role is to preserve the special ionic milieu of the inner ear fluids, specifically the potassium-rich endolymph and the sodium-rich perilymph, which is essential for the generation of the endocochlear potential (EP) and mechanical transduction by sensory hair cells.

Beyond its function in ionic homeostasis, the BLB serves as a highly selective barrier, meticulously controlling the passage of waste products and nutrients while limiting the entrance of most blood-borne cells, pathogens, and macromolecules. This selective permeability, which exhibits an inverse correlation with molecular size (molecules less than 100 Da cross more readily), significantly influences the specialized immune status of the cochlea (4). However, this barrier exhibits dynamic regulation rather than absolute impermeability. The integrity of the BLB can be compromised by several pathogenic disorders including acoustic trauma, inflammatory processes, ototoxic drugs like cisplatin and aminoglycosides, and acoustic trauma. Such disturbances can cause an influx of inflammatory mediators, immune cell invasion, and changes in inner ear fluid composition, which can either temporarily or permanently impair hearing (4).

Over the past decade, research has focused intensely on several key aspects concerning the BLB. These comprise studies on molecular mechanisms behind BLB disruption in different diseases, the development of more complex *in vitro* models to investigate their features, and the investigation of techniques to either transiently and safely alter the permeability for therapeutic drug delivery. Currently, novel non-invasive delivery strategies are under active investigation to bypass or temporarily open the BLB for enhanced therapeutic access; these include ultrasound combined with microbubbles, inner ear-targeting peptides, and even sound therapy.

The BLB presents a dual role in cochlear health and therapeutic intervention. Although its normal operation is crucial for preserving the delicate sensory structures and preserving the exact electrochemical gradients required for hearing, its constrictive character presents a major challenge for the systemic treatment agent delivery to the inner ear. Many cochlear pathologies are now understood to be related to a compromised BLB, which can be a consequence of the initial insult (e.g., noise, ototoxicity) and a factor that perpetuates additional damage by facilitating the uncontrolled influx of inflammatory cells and cytotoxic molecules (4). If precisely timed or if permeability can be transiently and controllably modulated, this pathological breach may ironically present a window of opportunity for therapeutic intervention. Therefore, a critical research direction involves developing methods to either protect and restore BLB integrity in conditions when its breakdown is detrimental or to induce a controlled, transient opening of the BLB to facilitate the targeted delivery of drugs for treating underlying cochlear diseases. The development of non-invasive techniques to achieve this modulation represents a significant advancement towards more effective inner ear therapies.

### 2.2 Resident immune cells of the cochlea

Rather than being an immunologically inert environment, the cochlea harbors a range of resident immune populations that are vital to homeostasis maintenance, damage response, and immune surveillance (5). A scoping review published in 2024 systematically collected data on these cells, confirming the presence of macrophages, lymphocytes, leukocytes, and mast cells in various inner ear structures of different mammalian species under steady-state conditions (**Table 1**). This underscores the need for further research on their specific roles (5).

**Table 1:** Key resident and recruited immune cells in the cochlea and their roles.

Cell type	Key markers/characteristics	Primary location(s) in cochlea	Roles in homeostasis	Roles in pathologies
Macrophages (General)	CD45+, CD68+, Iba1+, CX3CR1+	Stria vascularis, spiral ligament, Rosenthal’s canal, spiral ganglia, modiolus	Immune surveillance, debris clearance, tissue remodeling, and regulation of BLB permeability	CI: Fibrosis, response to implant material. ARHL: “Inflammaging,” chronic neuroinflammation, morphological/activation changes. NIHL/Ototoxicity: CX3CR1+ macrophages protective, involved in synaptic repair (via CX3CL1).
Perivascular Macrophage-like Melanocytes (PVM/Ms)	CD68+, MHC-II+, melanocyte markers	Stria vascularis (perivascular)	Antigen presentation, control of vascular permeability and contraction, and BLB regulation	Implicated in inflammatory responses, disruption can affect BLB integrity.
T Lymphocytes	CD3+, CD4+, CD8+	Found throughout cochlear tissues, numbers increase upon stimulation, endolymphatic sac	Immune surveillance, maintaining immune balance	AIED: Autoreactivity to cochlear antigens (e.g., cochlin). General: Can be pathogenic or protective depending on context and subset.
Dendritic Cells (DCs)	CD45+, CD11c+, MHC-II+	Stria vascularis, other cochlear tissues	Antigen uptake, processing, and presentation; initiation of adaptive immune responses; immune surveillance	AIED: Potential presentation of self-antigens. NIHL/Ototoxicity: Presentation of DAMPs/neoantigens. Upregulation of DC-associated genes in cochlear damage.
B Lymphocytes	CD19+, CD45+	Present in low numbers	Antibody production (potential for autoantibodies)	Limited specific data from 2020-2025; general role in humoral immunity.
Natural Killer (NK) Cells	CD45+	Present in low numbers	Cytotoxicity against infected or stressed cells	Limited specific data from 2020-2025; potential role in viral defense or early response to damage.
Granulocytes (e.g., Neutrophils)	CD45+, specific granule markers	Present in low numbers; they invade tissues during acute inflammation	Phagocytosis, release of inflammatory mediators	Limited specific data from 2020–2025 on steady-state roles; likely involved in acute inflammatory responses to infection or severe trauma.
Mast Cells	Specific granule markers (e.g., tryptase, chymase)	Various locations, often perivascular	Release of histamine, cytokines, and proteases; role in allergy and inflammation	Limited specific data from 2020–2025 on cochlear roles; general pro-inflammatory potential.

#### 2.2.1 Macrophages: the dominant sentinels and modulators

Under normal, homeostatic conditions, macrophages are the most common immune cell type found in the cochlea, accounting for approximately 81.3% of all CD45-positive immune cells (5). Their distribution is widespread across cochlear tissues, including the stria vascularis, the spiral ligament, Rosenthal’s canal (containing spiral ganglion neurons), and interspersed among the spiral ganglia themselves (6). A specialized subpopulation, known as perivascular melanocyte-like macrophages (PVM/Ms), resides within the stria vascularis and has been implicated in antigen presentation and the regulation of vascular permeability and contraction (7).

Research on cochlear macrophage populations has revealed variable expression patterns, detectable by markers such as CD68 and Ionized calcium-binding adapter molecule 1 (Iba1). These populations can exhibit different morphologies; for example, while CD68-expressing macrophages may be more round-shaped with a foamy appearance in some areas, Iba1-expressing macrophages often appear ramified or amoeboid. This morphological diversity most certainly reflects functional specialization. Cochlear macrophages are essential for immune surveillance, clearance of cellular debris, and may also help control BLB integrity and general tissue homeostasis under homeostatic conditions (6).

Under pathological conditions, macrophage function becomes more complicated and usually crucial. In the context of cochlear implantation (CI), after surgical trauma, macrophages are actively recruited and become part of the local immune response. They are found within fibrous sheaths that form around the electrode array and in fibrosis-affected tissues (8). Although in some cases their numbers may rise post-implantation, their exact contribution can be complex, as they both aid in wound healing and contribute to excessive fibrosis, which can compromise implant performance (8).

Macrophages undergo significant age-related changes in ARHL. While findings can exhibit variability between human studies and animal models, these include changes in morphology (e.g., a shift towards an amoeboid form suggestive of activation, reduced ramification), changes in their abundance and distribution within cochlear structures, and an overall increase in their activation state. This dysregulation is thought to be fundamental in “inflammaging”, a chronic, low-grade inflammatory condition that fuels neuroinflammation and cochlear tissue degeneration in ARHL (9).

In cases of ototoxicity and NIHL, macrophages exhibit a dual role. Resident macrophages expressing the chemokine receptor CX3CR1 have demonstrated protective capabilities by mitigating damage to hair cells resulting from invading immune cells (10). Repair of ribbon synapses between inner hair cells (IHCs) and spiral ganglion neurons depends on the CX3CL1 (fractalkine/CX3CR1) signaling axis, where CX3CL1 is expressed by neurons and IHCs and CX3CR1 by macrophages (11). On the other hand, deletion of CX3CR1 may exacerbate ototoxic drug-induced hair cell damage and hearing loss (12).

While a population of macrophages permanently resides in the cochlea (6), it is also well-established that circulating monocytes can be recruited and infiltrate cochlear tissue, especially under conditions of stress or damage, to augment the local immune response (13).

Cochlear macrophages represent a key therapeutic target, as their remarkable plasticity enables them to adopt different phenotypes and functions in response to the microenvironment and specific stimuli. Their ability to mediate both adverse effects, such as promoting fibrosis following cochlear implantation (8) or causing chronic inflammation in ARHL (9), as well as beneficial effects, such as enabling CX3CR1-dependent synaptic repair in NIHL, demonstrates this flexibility (14). Although not stated clearly for the cochlea in the given snippets, this functional dichotomy reflects the larger immunological idea of M1 (pro-inflammatory) and M2 (pro-resolving/reparative) macrophage polarization. Further supporting this context-dependent functional switching are the observed changes in macrophage shape and activation markers with age and damage. Future therapeutic interventions should therefore not aim for general macrophage suppression or depletion, as this could have counterproductive effects. Instead, treatments that selectively change macrophage phenotypes, promote pro-resolving activities, or suppress harmful pro-inflammatory activities are more likely to yield therapeutic effects. This comprehensive approach, potentially by targeting pathways like CX3CL1/CX3CR1 to enhance repair, is particularly vital for conditions where macrophage activity is a double-edged sword.

#### 2.2.2 T lymphocytes: modulators of adaptive and autoimmune responses

T lymphocytes, integral to adaptive immunity, are present in the cochlea, although their abundance is usually less than that of macrophages under normal, steady-state conditions (5). A systematic review published in 2025 showed that cochlear T cell populations can increase under different stimuli, suggesting either local proliferation during active immune responses or recruitment (15).

T cells are widely thought to exert diverse influences on cochlear health and disease. The existing literature emphasizes the delicate immune balance required to maintain cochlear homeostasis, describing T cells as having both negative roles that contribute to hearing loss and protective functions. This inherent duality suggests that distinct subsets of T cells, or T cells activated under varying conditions, can elicit opposing effects on auditory function.

A significant area of investigation pertains to the involvement of T cells in AIED. Cochlin, a protein primarily found in the inner ear, has been identified as a key autoantigen. Studies on T cell responses targeting cochlin have demonstrated its role in mediating Experimental Autoimmune Hearing Loss (EAHL) in animal models. Within the framework of human artificial intelligence, the development of antibodies and immune complexes involving cochlin is hypothesized to cause vascular inflammation, tissue damage, and progressive hearing loss (16). Fundamental research on cochlin has highlighted its role as a potential autoantigen in immune-mediated inner ear disease, a finding supported by a recent systematic review (15).

The observed dual capacity of T cells to be both pathogenic and protective strongly suggests that a significant determinant of cochlear outcomes is the nature of the T cell response—defined by the specific subsets involved (e.g., cytotoxic T lymphocytes, various T helper subsets like Th1, Th2, Th17, or regulatory T cells (Tregs)) and their antigen specificity. For example, autoreactive T cells targeting cochlin and other inner ear proteins are highly likely to cause pathology in AIED. On the other hand, Tregs, which modulate excessively strong immune responses (15) or specific T helper subsets that encourage tissue repair and inflammation resolution, could mediate protective roles. The documented rise in T cell counts following stimulation confirms that the cochlea can indeed mount an adaptive immune response. Thus, a better knowledge of the specific T cell subsets, their cytokine profiles, and their antigen targets in various cochlear diseases is indispensable. More focused immunotherapies, such as those aiming to selectively inhibit pathogenic T cell responses (e.g., anti-cochlin responses in AIED) or promote the activity of protective T cell populations, offer a more refined approach than general immunosuppression. This knowledge could pave the way for such therapies.

#### 2.2.3 DCs: initiators of adaptive immunity

Highly specialized professional antigen-presenting cells (APCs), dendritic cells are now understood to reside in the cochlea, including in crucial areas like the stria vascularis. DCs express Major Histocompatibility Complex class II (MHC-II) molecules on their surface, which are essential for presenting processed antigens to CD4^+^ T helper cells, thereby initiating and shaping adaptive immune responses (17).

Dendritic cells serve as crucial immune sentinels within tissues, continuously sampling their microenvironment for foreign antigens or danger signals. Upon encountering such stimuli, DCs undergo maturation and activation. This process involves upregulating chemokine receptors and co-stimulating molecules that help them migrate to draining lymph nodes, where they can effectively prime naive T cells and coordinate the ensuing adaptive immune response (18). Studies have demonstrated that genes linked to both macrophages and dendritic cells, including CD68 and MHC class II genes, are upregulated in the stria vascularis in animal models of cochlear damage, including the Slc26a4 knockout mouse, providing compelling evidence supporting their active role in the cochlea. Moreover, recent general immunology studies, including the discovery of GRASP55’s involvement in the trafficking of peptide-loaded MHC molecules to the DC surface (19), offer an understanding of the fundamental processes of antigen presentation by DCs, which are highly relevant to their possible purposes within the cochlear environment.

Given their robust antigen-presenting capacity and strategic location within the cochlea, DCs are exceptionally well-suited to act as immunological gatekeepers. They could either initiate immune responses against danger-associated molecular patterns (DAMPs) and neoantigens released during tissue damage from insults such as noise exposure or ototoxic drugs or against cochlear self-antigens (which might lead to AIED). This function is supported by the presence of DCs in cochlear tissues and the noted rise in their activity markers during cochlear damage. In conditions like AIED, where T cell responses to self-antigens, such as cochlin, are linked, DCs could be the primary cell type responsible for capturing, processing, and presenting these autoantigens to T cells, thereby triggering the autoimmune cascade. In response to acute cochlear damage, DCs may also process and display cellular debris or stress-induced proteins, contributing to the generation of an inflammatory milieu. This important function implies that targeting DC maturation, their antigen uptake mechanisms, or the co-stimulatory signals they provide to T cells could represent a novel therapeutic strategy to prevent or mitigate detrimental immune responses across a range of cochlear pathologies.

#### 2.2.4 Other immune cell populations

Although macrophages represent the predominant type of immune cell, under steady-state conditions, the cochlea also harbors other immune cell populations, albeit in smaller abundance. Flow cytometry studies have found B cells (about 0.4% of CD45-positive cells), granulocytes (about 3.1%), and natural killer (NK) cells (about 3.4%) (13) within the cochlea. Furthermore, a broader scoping review has noted the presence of various leukocytes and mast cells in the inner ear structures of mammals. Although the specific roles and contributions of these minority populations to cochlear immunity and pathology have been less extensively detailed in the literature over the past five years compared to macrophages and T cells, their presence indicates that they are part of the cochlear immune milieu. These cells may have specialized functions in response to different cochlear insults, at various stages of the disease, or during specific types of immune responses. More study is warranted to fully clarify their roles in cochlear health and disease.

## 3 Immune dysregulation in cochlear pathologies: recent insights

It is well-established that dysregulation of the finely tuned cochlear immune system represents a central mechanism in various auditory and vestibular pathologies. Over the past five years, research has shed fresh light on how immune responses start and progress these disorders (**Table 2**).

**Table 2:** Overview of immune-mediated cochlear pathologies.

Pathology	Key immunological features/mechanisms	Diagnostic advancements	Therapeutic developments
Autoimmune Inner Ear Disease (AIED)	Molecular mimicry, bystander effect; autoantibodies to HSP70, cochlin, collagen type II/IX; T-cell responses to cochlin; role of endolymphatic sac; primary or secondary to systemic disease.	Challenges persist (lack of specific biomarkers). Evaluation of anti-HSP70, aPL, ANA, and ESR. Advanced MRI (gadolinium-enhanced, 3D-FLAIR) for inflammation (non-specific). Genetic assessments (HLA, IL-1R polymorphisms). NIH initiatives.	Corticosteroids (systemic, intratympanic) remain first-line. DMARDs (methotrexate) and biologics (anti-TNF: infliximab, etanercept; IL-6R antagonists, rituximab) for refractory cases or as steroid-sparing agents; variable efficacy, need for RTCs. Personalized approaches based on immunologic profiles considered.
Cochlear Implantation (CI) Inflammation	Foreign body response to electrode; macrophage (CD68+, Iba1+) infiltration and activation; fibrosis/scar tissue formation around electrode; individual variability in inflammatory response.	The CHIEF study characterized the inflammatory state in pediatric CI. Histopathological analysis of macrophage distribution performed in implanted human temporal bones.	Corticosteroids (systemic, local/intracochlear/intratympanic) to reduce inflammation and fibrosis, improve impedance. Research into more biocompatible electrode materials and drug-eluting electrodes.
Noise-Induced Hearing Loss (NIHL)	ROS production, oxidative stress; activation of NF-κB, TNF, IL-17, TLR, and chemokine signaling pathways; upregulation of Relb, Ccl2, Ptgs2, Ccl17; immune cell infiltration; CX3CL1/CX3CR1 axis critical for synaptic repair and inflammation modulation.	Transcriptome analysis identifying key inflammatory pathways and molecules. Preclinical models demonstrating roles of specific immune cells and mediators.	Antioxidants (ALA, ebselen, Vit B12) and anti-inflammatories (Mg-aspartate, carbogen) in clinical trials (mixed results). Preclinical success with local sFKN (CX3CL1) delivery. Emerging strategies: Vagus Nerve Stimulation (VNS).
Age-Related Hearing Loss (ARHL)	“Inflammaging”; chronic low-grade inflammation; macrophage dysregulation (morphological changes, increased activation); elevated CRP, TNF-α, IL-1β, C3, C1q; microglial activation in auditory centers; CX3CL1/CX3CR1 axis involvement.	Clinical studies correlating inflammatory biomarkers with ARHL. Animal models demonstrating age-related changes in immune cells. Mendelian randomization studies exploring causality (genetic predisposition to inflammation may not be directly causal).	Targeting aging pathways: AMPK activators (metformin, resveratrol), sirtuin activators (NAD+ precursors), senolytics/senomorphics. Strategies to reduce cochlear oxidative stress and inflammation.
Meniere’s Disease	Potential autoimmune association (links to systemic autoimmune diseases); endolymphatic hydrops. Pilot studies suggest altered cytokine profiles (↑TNF-α, ↑IFN-γ, ↓ENA-78 in PBMCs).	Exploration of inflammatory markers (cytokines) to differentiate from other vestibular disorders. MRI for endolymphatic hydrops (not specific to the immune mechanism).	Primarily symptomatic treatment. Immunosuppression (corticosteroids) is sometimes used empirically, especially if AIED is suspected. Role of specific immunomodulators under investigation.

### 3.1 AIED

An unusual but important inflammatory disorder compromising the inner ear is AIED. Often bilateral but can start unilaterally, it is defined by fast progressive sensorineural hearing loss and may be accompanied by vestibular problems including tinnitus or dizziness. Less than 5 per 100,000 people are thought to be involved yearly (20). AIED can present as a primary disorder, wherein the pathology is limited to the inner ear, or as a secondary condition accompanying systemic autoimmune diseases, including rheumatoid arthritis, systemic lupus erythematosus (SLE), Cogan syndrome, granulomatosis with polyangiitis, and others (20).

#### 3.1.1 Pathogenesis – recent advances

Although the fundamental immunopathological processes underlying AIED are complex and not entirely clear, recent studies support hypotheses involving molecular mimicry, where immune responses to foreign antigens cross-activate similar inner ear self-antigens, and the “bystander effect”, where local inflammation non-specifically activates autoreactive immune cells (21).

A significant area of progress has involved the identification and characterization of specific autoantigens within the inner ear:

Heat Shock Protein 70 (HSP70), also known as the 68 kilodalton (kD) protein: Antibodies against HSP70 are frequently detected in AIED patients and have been investigated as potential diagnostic biomarkers (22). One study highlighted in a 2025 preprint reported anti-HSP70 antibodies demonstrating high sensitivity (79.07%) and specificity (100%) in AIED cases. However, a systematic review cited in the same preprint concluded that there is currently insufficient evidence to recommend routine diagnostic testing for HSP70 autoantibodies.

Cochlin: This protein, highly expressed in the inner ear, is emerging as a significant autoantigen in AIED. Research indicates that T-cell-mediated immune responses against cochlin can induce EAHL in animal models (15). Immune complexes involving cochlin are thought to contribute to cochlear damage and hearing loss in AIED patients, with antibodies targeting cochlin also playing a major role.

Other Antigens: Antibodies against other inner ear components, such as collagen type II and type IX, have also been identified in AIED patients, suggesting a broader autoimmune response against structural proteins of the cochlea (21).

Role of the Endolymphatic Sac: The endolymphatic sac, a structure within the inner ear possessing immunological functions, is considered a potential site for the generation and perpetuation of immune responses against self-antigens, particularly following an initial injury or inflammatory insult that might expose these antigens to the immune system (21).

#### 3.1.2 Diagnostic approaches and challenges

The diagnosis of AIED remains a significant clinical challenge. This challenge stems from the lack of a universally accepted gold standard diagnostic test, the significant overlap of its clinical symptoms (progressive hearing loss, vestibular symptoms) with other, more common causes of SNHL, and the absence of clear, specific biomarkers. Although response to steroid treatment is a common diagnostic criterion, its specificity is limited, and patient reactions exhibit considerable variability (20).

Recent advancements in diagnostic approaches include:

Laboratory Tests: The ongoing evaluation of anti-HSP70 antibodies persists, though their routine use is debated. Testing for antiphospholipid antibodies (aPL) may be relevant if an associated antiphospholipid syndrome is suspected. Standard immunological markers such as antinuclear antibodies (ANA), erythrocyte sedimentation rate (ESR), and rheumatoid factor (RF) are often assessed, particularly to screen for underlying systemic autoimmune conditions (20).

Imaging Techniques: Advanced Magnetic Resonance Imaging (MRI) techniques, such as intratympanic gadolinium-enhanced MRI and gadolinium (Gd)-enhanced MRI using 3D fluid-attenuated inversion recovery (FLAIR) sequences, have shown potential in visualizing inner ear structures and detecting inflammatory changes. While cochlear enhancement on MRI can indicate inner ear inflammation consistent with AIED, this finding is not specific to the disease and can be seen in other inflammatory conditions.

Genetic Assessments: There is growing interest in identifying genetic susceptibility factors for AIED. Certain Human Leukocyte Antigen (HLA) haplotypes (e.g., HLA-B27, B35, B51, C4, C7, A1-B8-DR3) and genetic polymorphisms in genes such as the Interleukin-1 Receptor (IL-1R) have been investigated for their potential roles as prognostic or susceptibility markers (20).

Aiming to promote research to better understand its pathogenesis and to build effective strategies for prevention and treatment, the National Institute on Deafness and Other Communication Disorders (NIDCD) and the Office of Autoimmune Disease Research (OADR) at the National Institutes of Health (NIH) have identified AIED as an area of portfolio interest after recognizing the inherent challenges and the need for progress in this field.

AIED’s heterogeneity is highlighted by its array of potential autoantigens (such as HSP70 and cochlin), varied responses to conventional treatments, and both primary and secondary presentations. A major obstacle to diagnosis, as well as the development of broadly effective treatments, is inherent variability. This challenge is highlighted by the present dependence on non-specific diagnostic markers and the varied value of tests such as anti-HSP70. Thus, in AIED research, a critical path forward involves stratifying patients into more homogeneous subgroups based on their specific underlying immunopathology, such as the type of immune response (e.g., T-cell-mediated vs. antibody-mediated). The development of robust biomarkers capable of identifying these distinct subgroups is of paramount importance. Such biomarkers would not only improve diagnosis but also help to design individualized medicine strategies by allowing the prediction of treatment response. Supported by NIH programs (22), this focused approach promises to go beyond empirical corticosteroid treatment towards more customized and effective interventions.

### 3.2 Inflammatory sequelae of cochlear implantation

For cases with severe-to-profound SNHL, cochlear implantation represents a highly effective surgical intervention that directly stimulates the auditory nerve to restore hearing. However, the implantation process itself, which involves the insertion of an electrode array into the delicate cochlea, inevitably causes trauma and triggers an immune response characterized by inflammation and subsequent wound-healing cascades in which macrophages are rather important (8).

A common consequence of this immune response is the development of fibrous scar tissue (fibrosis) around the electrode array. While some degree of tissue encapsulation is a natural aspect of healing, excessively robust or aggressive inflammatory reactions can lead to significant fibrosis. This excessive fibrosis is detrimental as it can increase the electrical impedance between the electrode contacts and neural elements, thereby diminishing the quality and efficiency of electrical stimulation. Moreover, unchecked inflammation can directly damage surviving cochlear structures, potentially resulting in the loss of any residual hearing a patient may have had prior to implantation, ultimately leading to poorer overall hearing outcomes with the implant. Research has revealed both CD68-positive and Iba1-positive macrophage populations in the fibrous sheath encircling the CI path and within fibrotic zones in the scala tympani and scala vestibuli. Although results can vary, some studies have observed an elevated density of these macrophages in implanted cochleae relative to non-implanted controls. The precise function of these macrophages, whether they predominantly contribute to adverse fibrosis or also assist in tissue repair and integration, remains an active area of ongoing research (6).

A notable characteristic of the post-CI inflammatory response is individual variation (23). Not all patients exhibit the same reaction to the implant, and the factors precipitating an excessive inflammatory response remain to be fully elucidated. This individual variability emphasizes the need for research aimed at defining and predicting these reactions. For example, the Cochlear Implants and Inner Ear Inflammation (CHIEF) study is a cross-sectional study designed to collect tissue and fluid samples from children and young people who have undergone cochlear implantation, in order to define the inflammatory state of the ear and investigate its relationship with long-term hearing outcomes. Elucidating these unique inflammatory variations could help to improve clinical treatment and provide CI users with better, more consistent hearing results.

By its nature as a foreign material inserted into a sensitive biological environment, the cochlear implant electrode can be considered a chronic foreign body. This perspective aids in understanding the continuous immune response as a sustained interaction that influences long-term device efficacy, rather than just as an acute reaction to surgical trauma. Following implantation, the initial acute inflammation may evolve into a chronic foreign body reaction, marked by ongoing macrophage activity and the progressive development of fibrosis around the electrode array. This continuous process can cause gradual changes in electrode impedance, potentially leading to a decline in hearing performance over time. Therefore, plans meant to improve CI results have to go beyond perfecting surgical methods to reduce initial trauma. They ought to also cover strategies to control the long-term immune response to the implant. This can entail the creation of drug-eluting electrodes that are able to directly release anti-inflammatory drugs or pro-resolving mediators at the electrode-tissue interface. Furthermore, advancements in biomaterials may lead to less immunogenic and more biocompatible electrode surfaces, thereby reducing adverse tissue reactions. Moreover, the ability to predict an individual’s inflammatory predisposition, as targeted by studies such as CHIEF, could enable customized pre-operative or peri-operative immunomodulatory treatments to optimize the cochlear environment for the implant.

### 3.3 NIHL

NIHL is a commonly acquired form of sensorineural hearing loss resulting from exposure to loud noise. Beyond direct mechanical damage, a significant number of studies has emphasized the important role of inflammatory and immune reactions in NIHL pathophysiology (7). Reactive oxygen species (ROS), which in turn starts an inflammatory response, activates apoptotic (programmed cell death) pathways, causes DNA fragmentation, and finally results in the death of sensory hair cells and auditory neurons. Acoustic overexposure sets off a cascade of events within the cochlea (24).

Pathways of inflammatory signaling and key molecules: Following noise exposure, transcriptome studies of cochlear tissue have revealed the activation of several inflammatory signaling pathways. Among these are the Tumor Necrosis Factor (TNF) signaling pathway, the Interleukin-17 (IL-17) signaling pathway, the Nuclear Factor-kappa B (NF-κB) signaling pathway, the Toll-like receptor (TLR) signaling pathway, and many chemokine signaling pathways (25). For example, the activation of NF-κB results in the synthesis of pro-inflammatory cytokines, caspases (enzymes involved in cell death), and other pro-apoptotic molecules, leading to hearing loss. Ten specific genes, including Relb (a component of the NF-κB pathway), Ccl2 (Monocyte Chemoattractant Protein-1, MCP-1), Ptgs2 (Prostaglandin-Endoperoxide Synthase 2, also known as COX-2, an enzyme vital for prostaglandin synthesis), and Ccl17 (a chemokine) have been identified to be upregulated in NIHL and linked with these inflammatory processes (25).

Immune Cell Involvement: CX3CL1/CX3CR1 Axis: After acoustic trauma, immune cells infiltrate the cochlea. Responses to cytokines and broader immune reactions are considered crucial components in NIHL pathogenesis. A particularly significant recent discovery is the function of the chemokine fractalkine (CX3CL1) and its receptor CX3CR1. Inner hair cells and cochlear neurons express CX3CL1, while CX3CR1 is found on resident cochlear macrophages (11). This signaling axis is widely thought to be vital for neuroprotection and repair. Research on CX3CR1-positive resident macrophages has revealed that they can protect against damage driven by other infiltrating immune cells (10). Moreover, the CX3CL1/CX3CR1 pathway actively repairs ribbon synapses (essential for auditory signaling) and modulates the inflammatory response following noise trauma. Local delivery of a soluble form of CX3CL1 (soluble fractalkine, sFKN) has been shown in preclinical studies to be able to restore these synapses, raise hearing thresholds, and reduce cochlear inflammation in a macrophage-dependent fashion. On the other hand, genetic disturbance of this pathway or depletion of CX3CR1-expressing macrophages can hinder synaptic repair and increase SGN loss and inflammation following noise trauma (14).

Growing evidence suggests that inflammation is not only a side effect of noise-induced damage but also a central and maybe modifiable factor of NIHL pathogenesis. This understanding facilitates the integration of complex biological reactions beyond purely mechanical damage. The identification of specific inflammatory pathways (such as TNF, NF-κB, and IL-17 signaling) (25) and mediators (like ROS, different cytokines, and chemokines) (24) activated by noise exposure opens new avenues for pharmacological intervention. Targeting these inflammatory pathways to either prevent or treat NIHL has therapeutic potential, as demonstrated by the encouraging outcomes of preclinical models employing antioxidants, anti-inflammatory drugs, or tailored immunomodulators such as sFKN (24). The success of sFKN in promoting synaptic repair and reducing inflammation in animal models is a particularly compelling illustration of how immunomodulatory strategies might be leveraged for hearing protection and restoration.

### 3.4 ARHL

ARHL, also known as presbycusis, is the most prevalent sensory disability among the elderly, characterized by a progressive, typically bilateral decline in hearing sensitivity, especially at higher frequencies (1). This condition entails degenerative changes in the central auditory paths and the peripheral cochlea. Recent studies have reported “inflammaging” a phenomenon characterized by persistent, low-grade inflammation, plays a significant role in the onset and advancement of ARHL (26).

Macrophage Dysregulation in ARHL: With aging, cochlear macrophages undergo remarkable changes, including alterations in their morphology (e.g., becoming more amoeboid, less ramified), abundance and distribution within cochlear tissues, and a general increase in their activation state. It is hypothesized that this age-associated macrophage dysregulation contributes to the chronic neuroinflammatory environment and tissue degradation seen in ARHL (9). The CX3CL1/CX3CR1 chemokine axis, which is crucial in NIHL, is widely thought to be relevant in ARHL since CX3CL1 gene expression is upregulated in the aging mouse cochlea, potentially influencing macrophage activity (11).

Cytokines and Complement System: Elevated systemic and local levels of inflammatory markers are associated with ARHL. These comprise components of the complement system, including C3 and C1q (26). C-reactive protein (CRP), pro-inflammatory cytokines including Tumor Necrosis Factor-alpha (TNF-α) and Interleukin-1 beta (IL-1β). For instance, while increased IL-1β is linked to synapse loss and worsening hearing thresholds in ARHL models, TNF-α can be neurotoxic at high concentrations and impair mitochondrial function in the aging ear. Interestingly, Mendelian randomization studies have indicated that although these inflammatory markers are elevated, a genetic inclination to systemic chronic inflammation may not, by itself, directly cause ARHL. This suggests that the age-related inflammatory processes in the auditory system may be driven more directly by local cochlear factors or the cumulative impact of environmental stresses over a lifetime.

Microglial activation in auditory centers: Beyond the cochlea, inflammation in the central auditory pathways also contributes to ARHL. Studies in aged mice have revealed heightened activation of microglia, the resident immune cells of the central nervous system, as indicated by increased expression of markers such as Iba1 and CD16, a marker of M1 microglial activation, in the cochlear nucleus. This central neuroinflammation further exacerbates hearing loss.

Although ARHL has been associated with inflammation, the relationship is complex. ARHL is widely believed to result from a complex interaction between intrinsic aging processes within the cochlea, the development of a local “inflammaging” state, and the cumulative effects of environmental factors and stresses throughout life; genetic predisposition to systemic inflammation alone does not appear to be the primary driver. The observation that cochlear macrophages exhibit pro-inflammatory changes with age, coupled with the finding that systemic genetic inflammatory tendencies may not directly correlate with ARHL risk, suggests that the cochlear-specific inflammatory milieu or the organ’s response to a lifetime of local stressors is particularly critical. This understanding suggests that rather than relying solely on systemic anti-inflammatory treatments, successful ARHL interventions may need to target these local cochlear inflammatory and aging pathways (27). Furthermore, the influence of lifestyle and environmental factors in modulating cochlear inflammaging warrants more extensive investigation to identify potential preventive strategies.

### 3.5 Meniere’s disease

Meniere’s disease is an inner ear disorder characterized by a classic triad of symptoms: episodic vertigo, fluctuating sensorineural hearing loss (typically affecting the low frequencies initially), and tinnitus or aural fullness. The precise etiology of Meniere’s disease remains elusive, though endolymphatic hydrops is a consistent histopathological finding. Notably, not all individuals with hydrops develop Meniere’s disease (28).

Potential Immunological Link: There is growing interest in the potential role of the immune system and inflammation in the pathophysiology of Meniere’s disease. Several autoimmune diseases, including rheumatoid arthritis, systemic lupus erythematosus, and ankylosing spondylitis, have been associated with Meniere’s disease, suggesting a possible shared autoimmune predisposition or pathogenic mechanism in a subset of patients (28). Some studies have also proposed a role for IgE in some instances.

Inflammatory Markers and Cytokine Profiles: Recent pilot studies have begun to explore specific inflammatory markers in Meniere’s disease. A study published in late 2024 compared proinflammatory cytokine profiles in patients with Meniere’s disease with those in patients with vestibular migraine and controls. Patient-derived peripheral blood mononuclear cells (PBMCs) from Meniere’s disease patients, when stimulated *in vitro*, tended to release higher levels of TNF-α and Interferon-gamma (IFN-γ), and lower levels of Epithelial Neutrophil-Activating Peptide 78 (ENA-78, also known as CXCL5), compared to vestibular migraine patients (29). These findings, though preliminary, suggest that distinct inflammatory pathways might contribute to Meniere’s disease.

Distinct cytokine profiles (elevated TNF-α and Interferon-gamma (IFN-γ), reduced ENA-78) have been observed in Meniere’s disease patients compared to those with vestibular migraine. Given that these two conditions can present with overlapping symptoms such as vertigo and auditory disturbances, identifying differential immune signatures could be of significant diagnostic value. If validated in larger cohorts, these immune markers may help differentiate between these disorders, which currently rely heavily on clinical criteria and the exclusion of other causes (29). This differentiation is crucial as it could lead to more targeted therapeutic approaches. For instance, if a specific inflammatory profile is consistently associated with Meniere’s disease, it could identify a subgroup of patients more likely to benefit from immunomodulatory treatments, potentially offering a new avenue for managing this challenging condition. Further research is warranted to confirm these immune signatures and understand their pathogenic role in Meniere’s disease.

## 4 Key inflammatory mediators in cochlear pathophysiology

The immune response within the cochlea, whether protective or pathological, is mediated by a complex interplay of soluble factors. Among these, cytokines, chemokines, and lipid mediators, such as prostaglandins, play pivotal roles in cell signaling, immune cell recruitment, and inflammatory modulation.

### 4.1 Cytokines: the messengers of inflammation and immunity

Cytokines are small proteins that act as critical intercellular messengers, regulating the intensity and duration of immune responses. An imbalance in their production or signaling can lead to aberrant immune cell activity and the establishment of a chronic pro-inflammatory microenvironment, ultimately causing damage to inner ear structures and function (11).

#### 4.1.1 Pro-inflammatory cytokines

Proinflammatory cytokines such as IL-6, IL-1β, and TNF-α are rapidly induced in the cochlea following injury, as first demonstrated by Fujioka et al. (2006), and have since been shown to play pivotal roles in both tissue damage and repair (30).

TNF-α: This potent cytokine has been implicated in several cochlear pathologies. In NIHL, the TNF signaling pathway is reportedly significantly activated (25). Similarly, ARHL has been associated with elevated TNF-α levels, which can be neurotoxic at high concentrations, impair mitochondrial function, and contribute to cellular damage. Pilot studies also suggest potentially higher levels of TNF-α in Meniere’s disease (29).

IL-1β: IL-1β represents another major pro-inflammatory cytokine. In animal models of ARHL, increased IL-1β has been associated with synapse loss between hair cells and auditory neurons, as well as progressive auditory threshold elevation (10). Furthermore, genetic polymorphisms in the IL-1 receptor have been linked to individual susceptibility to sudden sensorineural hearing loss (SSNHL), indicating a role for IL-1β activity in hearing impairment. As a pleiotropic inflammatory mediator, IL-1β participates broadly in inflammatory responses (31).

Interleukin-17 (IL-17): The IL-17 signaling pathway has been documented in the pathogenesis of NIHL (25), suggesting a role for Th17 cells or other IL-17-producing cells in noise-induced cochlear inflammation.

IFN-γ: Elevated levels of this cytokine, known for its role in Th1 responses and macrophage activation, have been observed in preliminary studies of Ménière’s disease patients (31).

#### 4.1.2 Anti-inflammatory and regulatory cytokines

Pro-inflammatory cytokines drive damage, while anti-inflammatory and regulatory cytokines are crucial for resolving inflammation and restoring homeostasis. Interleukin-10 (IL-10) and Transforming Growth Factor-beta (TGF-β) are well-known anti-inflammatory cytokines involved in the resolution phase of inflammation (31). More specifically, within the cochlea, therapeutic administration of sFKN in NIHL models has been shown to increase the levels of anti-inflammatory cytokines such as IL-10, IL-22, and IL-33, alongside its reparative effects.

The intricate balance between pro- and anti-inflammatory cytokines is critical for cochlear health. The outcome of an immune response is not solely dictated by the presence of a single cytokine, but rather by the overall cytokine milieu and the complex interplay within these signaling networks. For example, while low concentrations of TNF-α might facilitate hair cell survival through NF-κB activation, high concentrations can lead to apoptosis (11). This highlights that therapeutic strategies targeting cytokines may need to be more sophisticated than simply blocking a single pro-inflammatory mediator. A more comprehensive approach, aimed at restoring the appropriate balance, perhaps by selectively inhibiting key pathogenic cytokines, promoting the production or action of anti-inflammatory/regulatory cytokines, or targeting upstream regulators of these networks, could prove more effective and may yield fewer off-target effects.

### 4.2 Chemokines: guiding immune cell traffic

Chemokines represent a family of small proteins that play a fundamental role in directing the migration of immune cells to specific locations within the body, both during normal immune surveillance (homeostasis) and in response to inflammation or injury (11). They bind to G protein-coupled receptors on target cells, triggering intracellular signaling pathways that lead to cell movement and activation (32, 33).

#### 4.2.1 The CX3CL1/CX3CR1 axis

This chemokine system has emerged as a critical regulator of immune responses and neuro-immune interactions within the cochlea.

Expression and Function: CX3CL1, also known as fractalkine, is expressed by neurons, including spiral ganglion neurons (SGNs) and IHCs in the cochlea. Its unique receptor, CX3CR1, is primarily expressed on macrophages and microglia (11, 34, 35). This ligand-receptor pairing facilitates direct communication between neuronal and sensory cells, as well as immune cells.

Role in NIHL and Ototoxicity: The CX3CL1/CX3CR1 axis is vital for cochlear protection and repair. In the context of NIHL, intact fractalkine signaling is involved in the spontaneous repair of noise-damaged ribbon synapses (14). Local administration of soluble CX3CL1 has been shown to restore these synapses, improve hearing, and attenuate cochlear inflammation in a macrophage-dependent manner in animal models of NIHL. Conversely, genetic deletion of *Cx3cr1* or depletion of CX3CR1-expressing macrophages impairs synaptic repair and exacerbates SGN loss and inflammation following noise trauma. Similarly, *Cx3cr1* deletion worsens hearing loss and increases hair cell destruction in models of aminoglycoside ototoxicity (12). Overall, FKN signaling appears to be neuroprotective and anti-inflammatory in the cochlea.

Role in ARHL: Fractalkine gene transcripts are produced in human SGNs, spiral lamina regions, and the basilar membrane (36). Gene expression of FKN is upregulated in the aging mouse cochlea, suggesting its involvement in modulating macrophage activity during the aging process.

#### 4.2.2 Other chemokines

CCL2 (Monocyte Chemoattractant Protein-1, MCP-1): This chemokine is known to recruit monocytes/macrophages and microglia to sites of inflammation (37, 38). Its gene expression is upregulated in the mouse cochlea during normal aging and is also implicated as a key upregulated molecule in NIHL pathogenesis (39).

CXCL10: This chemokine has been shown to be upregulated in the cochlea following noise exposure (40).

ENA-78 (CXCL5): Preliminary studies suggest that levels of ENA-78 might be lower in the peripheral blood mononuclear cells of Meniere’s disease patients compared to those with vestibular migraine (29).

General Chemokine Signaling: The chemokine signaling pathway has been established as an important pathway involved in the pathogenesis of NIHL (41).

The consistent and significant role of the CX3CL1/CX3CR1 axis in modulating cochlear immune responses, particularly in the context of injury and repair processes such as synaptic regeneration, positions it as a prime therapeutic target (11). The expression of CX3CL1 by vulnerable cochlear cells (neurons, hair cells) and its receptor CX3CR1 by macrophages provides a direct communication line that influences macrophage recruitment and activity. Experimental evidence has demonstrated that disrupting this pathway can exacerbate damage in both ototoxicity and NIHL models. Conversely, enhancing it through sFKN administration promotes repair and reduces inflammation in NIHL, strongly suggesting its therapeutic potential. Modulating the CX3CL1/CX3CR1 axis, perhaps through the sFKN supplementation or small-molecule CX3CR1 agonists/positive allosteric modulators of CX3CR1 signaling, could offer a novel strategy for treating various forms of SNHL where macrophage activity and neuronal or synaptic damage are key features.

### 4.3 Prostaglandins and other lipid mediators

Lipid mediators, including prostaglandins (PGs) and leukotrienes (LTs), are powerful signaling molecules derived from fatty acids (primarily arachidonic acid) that play complex roles in inflammation and homeostasis (31).

Role in Inflammation: Prostaglandins, such as PGD2 and PGE2, and various leukotrienes are rapidly generated at sites of inflammation, acting as potent pro-inflammatory mediators that contribute to vasodilation, increased vascular permeability, pain, and fever. For instance, the early phase of inflammation is characterized by PGD2 upregulation (42). However, these roles exhibit considerable complexity; PGE2, for instance, demonstrates context-dependent biological activity, displaying either anti-inflammatory or pro-resolving properties that are determined by specific receptor engagement and cellular microenvironmental factors.

Cochlear Blood Flow: The vasoactive properties of these lipid mediators can influence cochlear blood flow. Foundational studies establishing the physiological framework have indicated that PGE2 can increase cochlear blood flow, while the leukotriene LTC4 can decrease it, suggesting that imbalances in these mediators could contribute to SNHL by affecting cochlear perfusion (43).

NIHL: The gene *Ptgs2*, which encodes cyclooxygenase-2 (COX-2), a key enzyme in prostaglandin synthesis, has been implicated in the pathogenesis of NIHL (44, 45). This suggests that increased prostaglandin production via COX-2 activity contributes to noise-induced cochlear damage.

Resolution of Inflammation: Crucially, the inflammatory process is not just about initiation and propagation but also involves an active resolution phase. Specialized pro-resolving mediators (SPMs), a class of lipid mediators derived from polyunsaturated fatty acids (including lipoxins, resolvins, protectins, and maresins), are endogenously produced and play a vital role in actively terminating inflammation, promoting the clearance of debris, and stimulating tissue repair and regeneration (31). The switch from pro-inflammatory eicosanoid production (like PGs and LTs) to SPM production is a key step in the resolution cascade.

A paradigm shift is emerging in therapeutic approaches to inflammation, transitioning from passive suppression to active promotion of resolution. While inflammation constitutes an essential physiological response to injury or infection, pathological persistence of inflammatory processes leads to chronic tissue damage and dysfunction (46–48). The cochlea, like other organ systems, maintains intrinsic resolution mediated by these SPMs. Traditional anti-inflammatory drugs, such as corticosteroids or non-steroidal anti-inflammatory drugs (NSAIDs) that inhibit prostaglandin synthesis (49–51), can be effective in reducing acute inflammation but may also have significant side effects with long-term use and might not fully restore tissue homeostasis or promote optimal healing. Therefore, therapeutic development is increasingly focusing on agents that mimic or enhance the production or action of SPMs. Such pro-resolution therapies could actively orchestrate the termination of cochlear inflammation and stimulate endogenous repair processes, potentially offering a more nuanced and effective approach with fewer adverse effects for conditions like NIHL, ARHL, or chronic inflammatory states in the inner ear.

## 5 Therapeutic strategies targeting cochlear immunity: progress and prospects

The growing understanding of the cochlear immune system’s role in various hearing disorders has spurred the development and investigation of therapeutic strategies aimed at modulating these immune responses. Over the past five years, significant progress has been made, encompassing the refinement of established immunosuppressive treatments and the exploration of novel immunomodulatory agents and regenerative approaches (**Table 3**).

**Table 3:** Immunomodulatory therapies for cochlear disorders: current and emerging approaches.

Therapeutic agent/strategy	Mechanism of action (brief, focusing on immune modulation)	Application in cochlear disorders	Key recent findings/clinical trial status
Corticosteroids (Systemic/Intratympanic)	Broad anti-inflammatory and immunosuppressive effects; inhibit cytokine production, immune cell trafficking and activation.	AIED, SSNHL, CI-related inflammation, Meniere’s Disease (if AIED suspected)	AIED: ~70% initial response, waning efficacy/relapse common.31 SSNHL: Combination therapy (systemic + IT) may be superior. CI: Reduce inflammation/fibrosis.8 IT methylprednisolone > IT dexamethasone for Meniere’s hearing.
Methotrexate	DMARD; inhibits dihydrofolate reductase, interfering with DNA synthesis and immune cell proliferation.	AIED (steroid-sparing)	Randomized trial showed no benefit as a steroid-sparing agent after initial prednisone response in AIED. Some case reports/series suggest utility.
Anti-TNF Biologics (e.g., Infliximab, Etanercept)	Target and neutralize TNF-α, a key pro-inflammatory cytokine.	AIED (refractory/steroid-sparing)	Variable results. Infliximab has shown promise in case reports, particularly in patients with concomitant inflammatory diseases (e.g., ulcerative colitis). Etanercept failed to show efficacy in one AIED trial. Need for larger RTCs.
Other Biologics (e.g., Rituximab, IL-6R antagonists)	Rituximab: Depletes CD20+ B cells. IL-6R antagonists: Block IL-6 signaling.	AIED (refractory/steroid-sparing)	Explored for AIED, limited data, primarily case reports/series. Potential for personalized therapy.
Ciclosporin/Tacrolimus (Calcineurin Inhibitors)	Inhibit calcineurin, preventing T-cell activation and cytokine production.	Inner Ear Stem Cell Therapy (immunosuppression)	Most common agents in animal models for cochlear stem cell transplantation; improve cell survival/migration. Recommended based on animal data and allied human trials.
Soluble CX3CL1 (sFKN)	Ligand for CX3CR1 on macrophages; promotes synaptic repair, modulates macrophage activity, anti-inflammatory.	NIHL (preclinical)	Local delivery restores ribbon synapses and hearing, attenuates inflammation in a macrophage-dependent manner in NIHL animal models. Potential immunotherapy for cochlear synaptopathy.
Antioxidants (e.g., ALA, Ebselen, Vitamins)	Scavenge ROS, reduce oxidative stress.	NIHL (prevention/treatment)	Clinical trials have shown mixed results. Partial efficacy: Mg-aspartate, carbogen, Vit B12, ALA, Ebselen (4kHz). Null results: NAC. Clinical significance and optimal regimen unclear.
Vagus Nerve Stimulation (VNS)	Modulates the autonomic nervous system; anti-inflammatory and anti-oxidative stress effects.	NIHL (emerging)	Known to reduce inflammation and oxidative stress. Preclinical studies show promise for tinnitus (related to NIHL). Potential to mitigate BLB disruption.
Senolytics/Senomorphics	Senolytics: Selectively eliminate senescent cells. Senomorphics: Suppress the harmful effects of senescent cells.	ARHL (prevention/treatment)	Preclinical research exploring targeting cellular senescence to mitigate ARHL. Part of broader strategy to target aging pathways.
Gene Therapy (Immunomodulatory Potential)	Delivery of genes encoding anti-inflammatory cytokines, Treg-promoting factors, or BLB-protective proteins.	AIED, NIHL, ARHL, support for Stem Cell Therapy (conceptual)	Primarily focused on correcting genetic defects for congenital HL. Immunomodulatory applications are largely conceptual but represent a future direction. Immune response to vectors is a consideration.

### 5.1 Immunosuppression in cochlear disorders

Immunosuppressive therapies, primarily corticosteroids, have long been the mainstay for conditions presumed to have an autoimmune or significant inflammatory component.

Corticosteroids: These remain the first-line treatment for AIED and are widely used for idiopathic SSNHL (3, 52).

Administration Route: Corticosteroids can be administered systemically (e.g., oral prednisolone, intravenous methylprednisolone) or locally via intratympanic injection (e.g., dexamethasone, methylprednisolone). Intratympanic delivery aims to achieve higher local concentrations in the inner ear while minimizing systemic side effects.

Efficacy and Limitations: In AIED, systemic corticosteroids achieve a response in approximately 60-70% of patients; however, responsiveness can diminish with prolonged use, and hearing loss may relapse upon tapering or discontinuation of the drug (22). For SSNHL, a network meta-analysis suggested that combination therapy (intratympanic plus systemic steroids) might offer the most significant improvement in hearing thresholds (23).

Use in Cochlear Implantation: Glucocorticoids are also employed in the context of CI to reduce acute inflammation and subsequent fibrosis associated with electrode insertion, which can positively impact electrode impedance and preservation of residual hearing. Both systemic and local (intracochlear or perioperative intratympanic) administration routes have been explored.

Disease-Modifying Antirheumatic Drugs (DMARDs) and Biologics: These agents are typically considered as steroid-sparing options or for patients with AIED who are refractory to corticosteroids.

Methotrexate: A randomized controlled trial found no benefit for methotrexate as a steroid-sparing agent in AIED patients who had initially responded to one month of prednisone (53). However, other reports and clinical experience suggest it may have some efficacy in some instances.

Anti-TNF Agents: Biologics targeting TNF-α, such as infliximab, etanercept, and adalimumab, have been investigated for AIED with variable results (54, 55). A case report highlighted the success of infliximab in a patient with AIED and co-existing ulcerative colitis, leading to hearing stabilization and cessation of oral steroid use. Other case reports also suggest potential benefits of infliximab. Conversely, a pilot placebo-controlled study of etanercept did not demonstrate efficacy over placebo in AIED patients (56).

Other Biologics: IL-6 receptor antagonists (57) and B-cell-depleting agents like rituximab are also being explored, primarily based on case series or small studies (58).

Challenges in Research: The rarity of AIED and the lack of standardized diagnostic criteria pose significant challenges to conducting large, robust randomized controlled trials (RCTs) for these biologic agents.

The heterogeneity in response to immunosuppressive treatments for AIED is a significant clinical challenge. The standard “one-size-fits-all” approach, typically starting with corticosteroids, often proves insufficient for long-term disease control or is limited by side effects. This variability strongly suggests that AIED is not a single, uniform disease entity. It can manifest as a primary condition, affecting only the inner ear, or as a secondary complication of various systemic autoimmune conditions, and likely involves different underlying autoimmune mechanisms and target antigens in different individuals. Biologic therapies, which target specific immune pathways (e.g., TNF-α, B cells), offer the potential for more directed treatment. The reported success of infliximab in an AIED patient with concomitant ulcerative colitis (56) underscores the possibility that matching the therapeutic agent to the patient’s specific immunologic profile or associated systemic inflammatory disease could be key to improving outcomes. Therefore, the future of AIED management will likely involve a transition towards personalized treatment strategies. This will require a better understanding of AIED subtypes, the development of reliable biomarkers to guide diagnosis and predict treatment response, and careful consideration of individual patient characteristics and comorbidities.

### 5.2 Immunomodulation for inner ear stem cell therapy

The advent of inner ear stem cell therapy represents a paradigm shift in treating SNHL, transitioning from augmenting existing neural structures with hearing aids or implants to regenerating damaged neural frameworks (23). However, a significant hurdle limiting the clinical translation of stem cell therapy is the host immune system’s potential rejection of donor cells.

Immunosuppressive Regimens in Translational Models: To address the challenge of immune rejection, various immunosuppressive strategies are being explored, primarily in animal models, given that human trials for inner ear stem cell therapy are not yet prevalent (23).

Ciclosporin: This calcineurin inhibitor has been the most frequently used immunosuppressive agent in animal studies of cochlear stem cell transplantation. Systemic administration (subcutaneous or intraperitoneal) of ciclosporin has been shown to increase the survival, integration, and migration of transplanted stem cells in species like guinea pigs, mice, and gerbils. Dosages have varied widely depending on the animal model.

Steroids: While extensively used for other inner ear pathologies, corticosteroids (e.g., dexamethasone) have been less systematically investigated in animal models specifically for stem cell therapy. Some studies have suggested that systemic administration—rather than local delivery—may be required for optimal efficacy, as systemic corticosteroids can modulate both cochlear inflammation and host immune responses to transplanted cells (59).

Necessity of Immunosuppression: Some studies indicate that systemic immunosuppression is crucial for the successful transplantation and survival of otic progenitor cells, particularly when using allogeneic or xenogeneic cells.

Cochlear Immune Privilege in the Context of Stem Cell Therapy: Interestingly, several animal studies involving intracochlear stem cell transplantation did not employ immunosuppression and reported no significant evidence of immune or inflammatory reactions in the short term. This suggests that the cochlea might possess a degree of immunological privilege that could be conducive to cell-based therapies. However, this is not a universal finding, as one study reported an immune response to transplanted human otic progenitor cells in guinea pigs even without overt rejection (23).

Recommendations and Future Directions: Based on current evidence from cochlear animal studies and drawing parallels from human stem cell trials in related fields (e.g., retinal and spinal tissues), calcineurin inhibitors like tacrolimus or ciclosporin are often suggested as potentially useful immunosuppressive agents. Steroids may also play a role, particularly during the peri-transplant period, in managing acute inflammation related to the surgical procedure.

The concept of cochlea immune privilege within the context of stem cell transplantation appears to be “conditional” rather than absolute. While the inner ear may offer a relatively protected environment compared to other systemic sites, this privilege can likely be overwhelmed, especially when dealing with allogeneic (same species, different individual) or xenogeneic (different species) stem cells. The type of stem cell used (e.g., mesenchymal stem cells, which have inherent immunomodulatory properties, versus more immunogenic pluripotent stem cell derivatives), the method of delivery, the degree of surgical trauma, and the underlying immune status of the host are all factors that likely influence the necessity and intensity of immunosuppression. The variability in outcomes in animal studies, with some demonstrating cell survival without immunosuppression and others indicating rejection or immune responses, underscores this complexity. Therefore, a deeper understanding of the specific immune responses elicited by different types of stem cells within the cochlear microenvironment is crucial. Future strategies will likely need to be tailored, potentially involving not only systemic immunosuppression but also local delivery of immunomodulatory factors, the use of less immunogenic cell sources (such as autologous cells or induced pluripotent stem cells (iPSCs) differentiated into otic lineages), or genetic engineering of stem cells to reduce their immunogenicity. Optimizing the timing, duration, and type of immunosuppression will be critical for the success of regenerative therapies for hearing loss.

### 5.3 Novel therapeutic avenues for NIHL and ARHL

Beyond established immunosuppressants, research is actively exploring novel therapeutic agents and strategies that target the specific immune and inflammatory mechanisms underlying NIHL and ARHL.

#### 5.3.1 NIHL

Antioxidants and Anti-inflammatory Agents: Given the roles of ROS and inflammation in NIHL (60, 61), various agents with antioxidant or anti-inflammatory properties have been investigated in clinical trials. These include N-acetylcysteine (NAC), alpha-lipoic acid (ALA), ebselen (an organoselenium compound with glutathione peroxidase-like activity), magnesium aspartate, and various vitamins (e.g., B12, C, E). A systematic review up to February 2020 found that while some agents showed promising results in reducing temporary or permanent threshold shifts (e.g., Mg-aspartate, carbogen, vitamin B12, ALA, and ebselen at 4 kHz), the overall clinical significance and optimal regimens remain unclear due to heterogeneity in study designs and methodologies (24). NAC, despite extensive preclinical investigation, did not show significant efficacy in the clinical trials included in that review (62).

Targeting Specific Pathways (e.g., CX3CL1/CX3CR1): Preclinical research has shown significant promise for therapies targeting specific molecular pathways. Local delivery of soluble fractalkine has been demonstrated to restore ribbon synapses, improve hearing, and attenuate cochlear inflammation in animal models of NIHL, an effect dependent on macrophages (14). This highlights the potential of targeted immunotherapies.

Vagus Nerve Stimulation (VNS): Emerging evidence suggests VNS, particularly transcutaneous VNS (tVNS), as a potential non-invasive therapy for NIHL. VNS is known to exert anti-inflammatory and anti-oxidative stress effects, which could counteract key pathological mechanisms in NIHL and associated BLB disruption (63).

#### 5.3.2 ARHL

Targeting Fundamental Aging Pathways: Therapeutic strategies for ARHL are increasingly focused on addressing the underlying molecular and cellular aging processes that contribute to cochlear degeneration and “inflammaging” (27). These include:

AMPK Activation: Modulating the AMP-activated protein kinase (AMPK) pathway through caloric restriction, or compounds like resveratrol and metformin, which can reduce oxidative stress and inflammation (64, 65).

mTOR Inhibition: Targeting the mammalian target of rapamycin (mTOR) pathway, a central regulator of cell growth and aging (66).

Sirtuin Activation: Enhancing the activity of sirtuins (e.g., SIRT1, SIRT3), proteins involved in cellular longevity, apoptosis, and inflammation, potentially through NAD+ precursors (67).

Cellular Senescence: Using senolytics or senomorphics to combat the accumulation of senescent cells in the aging cochlea (68).

Autophagy Enhancement: Modulating pathways like AMPK, mTOR, and sirtuins to improve autophagy, the cellular “housekeeping” process that declines with age.

Oxidative Stress Reduction: Strategies aimed at reducing ROS and mitochondrial damage.

Preventing Inflammaging: A key goal is to develop interventions that can mitigate the chronic, low-grade inflammation characteristic of the aging auditory system (26).

A striking overlap characterizes the pathophysiology of NIHL and ARHL, wherein chronic inflammation and oxidative stress emerge as central drivers of cochlear damage. NIHL is characterized by acute ROS production and inflammation involving pathways like NF-κB and TNF signaling (69, 70), while ARHL involves a more chronic “inflammaging” state with macrophage dysregulation and similar cytokine involvement (e.g., TNF-α, IL-1β). This overlap suggests a potential for cross-application of therapeutic strategies. Interventions that successfully mitigate inflammation and oxidative stress in one condition might prove beneficial for the other. For example, antioxidants explored for NIHL share common ground with therapies targeting oxidative stress and senescence in ARHL (27). Furthermore, understanding how repeated noise exposure might accelerate or exacerbate the “inflammaging” processes observed in ARHL could reveal synergistic risk factors and pave the way for combined or earlier preventive strategies.

### 5.4 Gene therapy

Gene therapy is emerging as a highly promising future therapeutic modality for SNHL, with an initial emphasis on congenital forms of hearing loss caused by single-gene defects. The fundamental principle of gene therapy is to introduce genetic material into target cells to replace or correct a defective or missing gene, thereby restoring normal cellular function and, in the context of hearing loss, potentially restoring auditory capabilities (71).

Several active clinical trials are currently underway, testing the safety and efficacy of gene therapy approaches for specific genetic forms of hearing loss. While no gene therapy for hearing loss has yet received FDA approval for routine clinical use, the progress in this field is rapid, fueled by advancements in vector technology (e.g., adeno-associated viruses, AAVs) and gene editing tools like CRISPR/Cas9. Recent news highlights include promising results in preclinical models for genetic deafness and early human trials (72–75). Damage to various cellular components in the cochlea, including hair cells and the ribbon synapses between inner hair cells and spiral ganglion neurons, can cause SNHL. Many genes associated with deafness have been identified in these structures, providing targets for gene therapy (76).

While the primary aim of many current gene therapies for hearing loss is to correct genetic defects, the intersection of gene therapy with cochlear immunology is an important consideration and a potential area for future development. The introduction of viral vectors or transgene products can itself elicit an immune response within the cochlea, potentially limiting the efficacy or durability of the therapy. Therefore, understanding and managing these host immune responses is crucial for the long-term success of cochlear gene therapy. Conversely, gene therapy also offers a powerful tool for actively modulating the cochlear immune environment to achieve therapeutic benefits. It could be engineered to deliver genes encoding anti-inflammatory cytokines (e.g., IL-10), factors that promote regulatory T cell development or function, proteins that enhance the integrity of the BLB, or neurotrophic factors that protect auditory neurons from inflammatory damage. Such immunomodulatory gene therapies could be beneficial not only for genetic forms of hearing loss with an inflammatory component but also for acquired conditions like AIED or to create a more favorable environment for the survival and integration of transplanted stem cells. Thus, the immunogenicity of gene therapy vectors needs careful evaluation and management, while the potential for gene therapy to deliver localized and sustained immunomodulation within the cochlea represents an exciting future direction.

## 6 Future directions and unanswered questions

Despite significant progress in understanding cochlear immunology over the past five years, numerous questions remain, and several key areas require focused research to translate current knowledge into effective clinical interventions for hearing loss.

Biomarker Discovery: A critical and urgent unmet need is the identification and validation of specific and reliable biomarkers for immune-mediated cochlear diseases, particularly AIED. Such biomarkers are essential for early and accurate diagnosis, predicting disease course, monitoring treatment efficacy, and stratifying patients for targeted therapies. The current lack of robust biomarkers significantly hampers clinical trial design and the development of personalized medicine approaches for these conditions.

Understanding Immune Cell Heterogeneity and Interactions: While the presence of key immune cell types, such as macrophages, T cells, and dendritic cells, in the cochlea is now established, a more comprehensive characterization of their specific subsets, activation states, and intricate interactions within the cochlear microenvironment in both health and disease is required. For instance, determining the distinct roles of M1 versus M2 macrophage phenotypes, as well as various T helper and regulatory T cell subsets, in different cochlear pathologies will be crucial for developing more precise immunomodulatory strategies.

Targeted and Personalized Immunomodulation: The future of treating immune-mediated hearing loss lies in moving beyond broad immunosuppression towards more targeted and personalized therapies. This involves developing interventions that can selectively modulate pathogenic immune pathways while preserving or even enhancing protective immune responses. Tailoring treatments based on individual patient immunologic profiles, specific autoantigens (e.g., in AIED), or genetic predispositions is a key goal.

Optimizing Drug Delivery to the Inner Ear: The BLB remains a formidable challenge for delivering therapeutics to the cochlea effectively and safely. Continued research into novel drug delivery systems, including nanotechnology-based carriers, methods to transiently and reversibly modulate BLB permeability (e.g., ultrasound-mediated delivery), and inner ear-targeting strategies, is vital for translating many promising therapeutic compounds into clinical practice.

Translational Research and Clinical Validation: Many exciting findings in cochlear immunology, such as the therapeutic potential of sFKN for NIHL or senolytics for ARHL (27), have emerged from preclinical studies. Rigorous and well-designed clinical trials are necessary to validate these findings in human patients and to determine their safety and efficacy. Bridging this translational gap is a major priority.

Long-term Effects of Cochlear Implantation: Further investigation is needed to understand the long-term immunological consequences of cochlear implantation, including the chronic inflammatory response to the electrode array and the mechanisms driving progressive fibrosis in some individuals (77). Developing strategies to mitigate these chronic effects could improve long-term implant performance and patient outcomes.

Role of the Microbiome: The influence of gut microbiome on systemic immunity and its links to various autoimmune and inflammatory diseases are well-established. Exploring the potential role of the microbiome (both gut and potentially local ear microbiota) in modulating cochlear immunity and susceptibility to hearing disorders is an emerging area that warrants investigation.

Investigating the Cochlear Lymphatic System: The recent discovery and characterization of a functional lymphatic system in the central nervous system (CNS) (78) has revolutionized neuroimmunology. While the cochlea has traditionally been thought to lack classical lymphatic drainage, further exploration into whether a similar, perhaps non-conventional, lymphatic or glymphatic-like clearance system exists in the inner ear could significantly impact our understanding of immune surveillance, antigen clearance, and fluid homeostasis in the cochlea.

## 7 Conclusion

The past five years (2020–2025) have witnessed substantial advancements in the field of cochlear immunology, transforming our understanding of the inner ear from a passively protected, immune-privileged organ to one possessing a dynamic and locally regulated immune system. This paradigm shift has profound implications for how we approach the management of hearing loss. It is now understood that the cochlea is equipped with a resident arsenal of immune cells, including macrophages, T lymphocytes, and dendritic cells, which, along with the critical blood-labyrinth barrier, actively participate in maintaining homeostasis and responding to a variety of insults.

Key insights from this period have underscored the complex roles these immune components play in the pathogenesis of diverse cochlear disorders. Macrophages emerge as multifaceted cells, capable of both contributing to damaging inflammation and fibrosis (as observed in ARHL and post-CI responses) and promoting repair and protection (as highlighted by the CX3CL1/CX3CR1 axis in NIHL). T cells, particularly in the context of AIED, can drive autoimmune responses against cochlear-specific antigens, such as cochlin; however, they also possess the potential for protective functions. Dendritic cells are strategically positioned to initiate adaptive immune responses, acting as crucial sentinels and potential instigators of autoimmunity or inflammation. The integrity and selective permeability of the BLB are now better understood as critical factors influencing both cochlear health and the feasibility of therapeutic interventions. Furthermore, specific inflammatory mediators, including a range of cytokines (TNF-α, IL-1β, IL-17), chemokines (CX3CL1, CCL2), and lipid mediators (prostaglandins, SPMs), have been identified as key players in the molecular dialogues that shape cochlear immune responses and contribute to the pathology.

These evolving understandings have direct therapeutic implications. While corticosteroids remain a cornerstone for acute inflammatory conditions like AIED and SSNHL, their limitations have spurred the investigation of more targeted approaches, including DMARDs and biologic agents, although robust clinical evidence for many of these is still forthcoming. For the burgeoning field of inner ear stem cell therapy, managing the host immune response through tailored immunosuppression, likely involving calcineurin inhibitors, is recognized as a critical challenge for successful cell engraftment and function. Moreover, novel therapeutic avenues are emerging for NIHL and ARHL, moving beyond symptomatic relief to target the underlying mechanisms of inflammation, oxidative stress, and cellular aging. Agents such as sFKN, VNS, and senolytics are showing promise in preclinical and early clinical stages. Gene therapy also holds future potential, not only for correcting genetic defects but possibly for delivering immunomodulatory factors directly to the cochlea.

Looking ahead, the field of cochlear immunology is poised for further breakthroughs. The urgent need for specific diagnostic and prognostic biomarkers, particularly for AIED, remains a priority. A deeper dissection of immune cell heterogeneity and their intricate interactions within the cochlear microenvironment will be essential for developing precision medicine strategies. Future research should further delineate the dynamic interplay between innate and adaptive immune responses within the cochlea and explore how non-immune cochlear cells, such as supporting and hair cells, contribute to inflammatory modulation. These aspects represent promising directions for targeted immunomodulatory therapies. Optimizing drug delivery to the inner ear, bridging the translational gap from bench to bedside, and understanding the long-term immune consequences of interventions like cochlear implantation are also critical areas of focus. Continued research into these complex immune mechanisms holds immense potential for revolutionizing the diagnosis, treatment, and ultimately prevention of a broad spectrum of hearing disorders, with the overarching goal of preserving and restoring the precious sense of hearing by harnessing and appropriately modulating the body’s immune system.
